# Recent Trends in Liver Cancer: Epidemiology, Risk Factors, and Diagnostic Techniques

**DOI:** 10.7759/cureus.72239

**Published:** 2024-10-23

**Authors:** Shivani R Kale, Geeta Karande, Anand Gudur, Aishwarya Garud, Monika S Patil, Satish Patil

**Affiliations:** 1 Molecular Biology and Genetics, Krishna Institute of Medical Sciences, Krishna Vishwa Vidyapeeth (Deemed to be University), Karad, IND; 2 Microbiology, Krishna Institute of Medical Sciences, Krishna Vishwa Vidyapeeth (Deemed to be University), Karad, IND; 3 Oncology, Krishna Institute of Medical Sciences, Krishna Vishwa Vidyapeeth (Deemed to be University), Karad, IND

**Keywords:** cirrhosis, early diagnosis, hbv and hcv epidemiology, hepatocellular carcinoma, intrahepatic cholangiocarcinoma

## Abstract

Liver cancer, particularly hepatocellular carcinoma (HCC), poses a significant global health challenge due to its high mortality rate. Hepatocellular carcinoma and intrahepatic cholangiocarcinoma (ICC) are the two main types of primary liver cancer (PLC), each with its own set of complexities. Secondary or metastatic liver cancer is more common than PLC. It is frequently observed in malignancies such as colorectal, pancreatic, melanoma, lung, and breast cancer. Liver cancer is often diagnosed at an advanced stage, making it difficult to treat. This highlights the need for focused research on early detection and effective treatment strategies.

This review explores the epidemiology, risk factors, and diagnostic techniques for HCC. The development of HCC involves various risk factors, including chronic liver diseases, hepatitis B and C infections, alcohol consumption, obesity, smoking, and genetic predispositions. Various invasive and non-invasive diagnostic techniques, such as biopsy, liquid biopsy, and imaging modalities like ultrasonography, computed tomography scans (CT scans), magnetic resonance imaging (MRI), and positron emission tomography (PET) scans, are utilized for HCC detection and monitoring. Advances in imaging technology and biomarker research have led to more accurate and sensitive methods for early HCC detection. We also reviewed advanced research on emerging techniques, including next-generation sequencing, metabolomics, epigenetic biomarkers, and microbiome analysis, which show great potential for advancing early diagnosis and personalized treatment strategies.

This literature review provides insights into the current state of liver cancer diagnosis and promising future advancements. Ongoing research and innovation in these areas are essential for improving early diagnosis and reducing the global burden of liver cancer.

## Introduction and background

Liver cancer, particularly hepatocellular carcinoma (HCC), is a deadly disease and remains a significant global health issue [1]. It is an aggressive tumor that often develops in individuals with chronic liver disease, although it can also occur in the absence of such conditions [2]. According to Global Cancer Statistics 2020, liver cancer is the sixth most commonly diagnosed cancer worldwide. It ranks as the third leading cause of cancer-related deaths, responsible for approximately 830,000 deaths in 2020 [3]. The prevalence of HCC is generally higher in males than females [4]. According to the World Health Organization (WHO), liver cancer is projected to bring over one million deaths in 2030 [5].

There are two main types of liver cancer, HCC and cholangiocarcinoma, also known as primary liver cancer (PLC) [6]. Hepatoblastoma and angiosarcoma are uncommon forms of liver cancer [7]. The process of HCC involves several steps. It arises from the main parenchymal cells of the liver, known as hepatocyte cells. These cells continually damage and regenerate, causing liver fibrosis and eventually increasing the risk of developing cirrhosis. Eventually, cirrhosis may progress to HCC and sometimes invade surrounding tissues and can cause metastatic occurrence [8]. The disease is identified at an advanced stage, so some current medicines are limited to palliative care and local therapy. However, cell cycle-based therapies offer promising new options, as they target the rapid proliferation of cancer cells, which contrasts with the predominantly resting state of healthy liver cells [9].

Cholangiocarcinoma, or bile duct cancer, is an epithelial cell malignancy that originates in the bile ducts from the liver to the gallbladder and small intestine. It is the second most prevalent primary liver malignancy after HCC and is categorized into three subtypes depending on anatomical location: Distal common carotid artery (dCCA) occurs in the bile duct near the small intestine, perihilar CCA (pCCA) occurs at the bile duct junction where it enters the liver, and intrahepatic CCA (iCCA) arises within the liver itself [10]. Intrahepatic cholangiocarcinoma (ICC) makes up about 10% of the cholangiocarcinoma. Combined HCC-ICC tumors tend to exhibit the more aggressive behavior characteristic. Moreover, ICC is typically diagnosed at advanced stages, either with metastatic spread or local invasion. Globally, ICC accounts for up to 20% of all cholangiocarcinoma cases, with an incidence of 0.85 per 100,000 people [11].

Secondary or metastatic liver cancer occurs when cancer cells from a primary malignancy, such as those originating in the colon, breast, or lungs, spread to the liver, resulting in metastatic lesions. These cancer cells metastasize to the liver through blood or lymphatic pathways and proliferate to form secondary liver cancer, contributing to disease progression [12]. The liver is the most common site for metastases originating from colorectal cancer (CRC). Over half of CRC patients develop metastases, with a majority of these involving the liver. The five-year survival rate following surgical resection of liver metastases ranges from approximately 24% to 40%. However, overall survival rates for colorectal liver metastases remain poor, particularly in patients with unresectable disease, highlighting the challenges in managing advanced-stage metastasis [13].

Each liver cancer type has a distinct etiology, histology, and treatment protocol, presenting challenges for accurate diagnosis and effective treatment planning. Analysis of HCC has shown abnormal expression or exertion of growth factors, receptors, cellular alterations, and related signaling pathways [10]. Risk factors vary widely across cases and regions, with key contributors including hepatitis B, hepatitis C, alcoholic cirrhosis, diabetes, and non-alcoholic steatohepatitis [14]. Hepatitis can lead to liver fibrosis and eventually, cirrhosis, which is the leading cause of death in affected individuals [15].

The treatment of HCC primarily focuses on surgical options like resection and liver transplantation, often limited by the disease's advanced stage at diagnosis. Radiofrequency ablation (RFA) has emerged as a key treatment option, particularly during the early stages of PLC. Additionally, locoregional and systematic therapies have significantly improved patient outcomes [16]. Recent studies have linked morphological features of HCC with inheritable genetic mutations and dysregulation of key natural biological pathways, such as Wnt/β-catenin and P53, which regulate cell growth and apoptosis. These inherited abnormalities and pathway disruptions can contribute to uncontrolled cell proliferation and tumor development in HCC [17]. Ongoing research is crucial for advancing survival rates and enhancing the quality of life for patients with HCC.

This review explores the evolving landscape of HCC, highlighting the key risk factors that contribute to the development of this deadly liver cancer and diagnostic techniques. Additionally, the review delves into the future outlook, examining emerging technologies and techniques that hold promise for enhancing personalized treatment strategies and improving patient outcomes.

## Review

Liver cancer ranks among the top five most deadly cancers in 90 countries, with varying global incidence rates [18]. Between 2005 and 2015, HCC was the second leading cause of cancer mortality worldwide, following lung cancer. During this period, there was a 4.6% increase in the absolute number of years of life lost due to liver cancer (95% confidence interval: 1.6% to 15.4%) [19]. According to the Global Cancer Observatory (GLOBOCAN) 2018 report, liver cancer is the third leading cause of cancer-related deaths worldwide, contributing to 781,631 deaths in 2018. Furthermore, the GLOBOCON 2020 report indicates that Mongolia has the highest age-standardized rates of both liver cancer prevalence and mortality. China still comprises the majority (62.4%) of cases in Asia, followed by Japan (7.0%), India (5.3%), Thailand (4.2%), and Vietnam (4%). Factors like hepatitis infections, alcohol consumption, and limited healthcare access drive the high liver cancer rates in Mongolia and other Asian countries. Effective public health measures, including vaccination, screening, and lifestyle interventions, are critical to reducing these rates [20,21]. Liver cancer rates seem to be steadily decreasing in Sri Lanka [22]. While many other major cancers are declining, liver cancer rates have been increasing globally, particularly between 2010 and 2019 [23]. Although global rates remained steady, they significantly rose in America [24]. Additionally, around one-third of individuals with cirrhosis are likely to develop liver cancer during their lifetime [25].

According to Global Cancer Statistics, in nearly all populations, males have more advanced liver cancer rates than females between the 21 and 41 age groups [26]. The disease is particularly prevalent in East and Southeast Asia, where viral hepatitis is widespread [27]. The age at which HCC appears varies according to gender, geographic area, and risk factors associated with cancer development [28]. A new study published in the Journal of Hepatology by scientists from the International Agency for Research on Cancer (IARC) assesses the global burden of liver cancer in 2020. It predicts that the annual number of new cases and deaths will rise by over 55% by 2040 [15]. This alarming projection highlights the urgent need for improved prevention, early detection, and more effective treatment strategies to combat this disease in the coming years.

Risk factors

The major risk factors for HCC differ by region [29]; HCC is driven by several key risk factors, including chronic hepatitis B and C infections, each contributing 1.5 million new cases annually. Chronic hepatitis B virus (HBV) affects 296 million people, while hepatitis C virus (HCV) impacts 58 million. Alcohol consumption is a major factor, particularly in Europe and the Americas, where 43% of the population drinks heavily. Non-alcoholic fatty liver disease (NAFLD) affects 32.4% of adults globally, with obesity and diabetes also contributing significantly. Aflatoxin exposure, cirrhosis, and hepatitis D virus (HDV) further increase HCC risk, underscoring its complex global epidemiology [30]. Also, metabolic dysfunction-associated fatty liver disease (MAFLD), non-alcoholic steatohepatitis (NASH), smoking, both genetic and non-genetic, and exposure to environmental carcinogens, co-infection with HIV alongside HBV or HCV, also elevate the risk due to impaired immune function and enhanced liver disease progression. Other viruses, such as human T-cell lymphotropic virus type 1 (HTLV-1) and Epstein-Barr virus (EBV), have also been associated with an increased risk of HCC in cases of co-infection [11, 31]. However, HCC can also occur in individuals without these risk factors.

**Figure 1:**
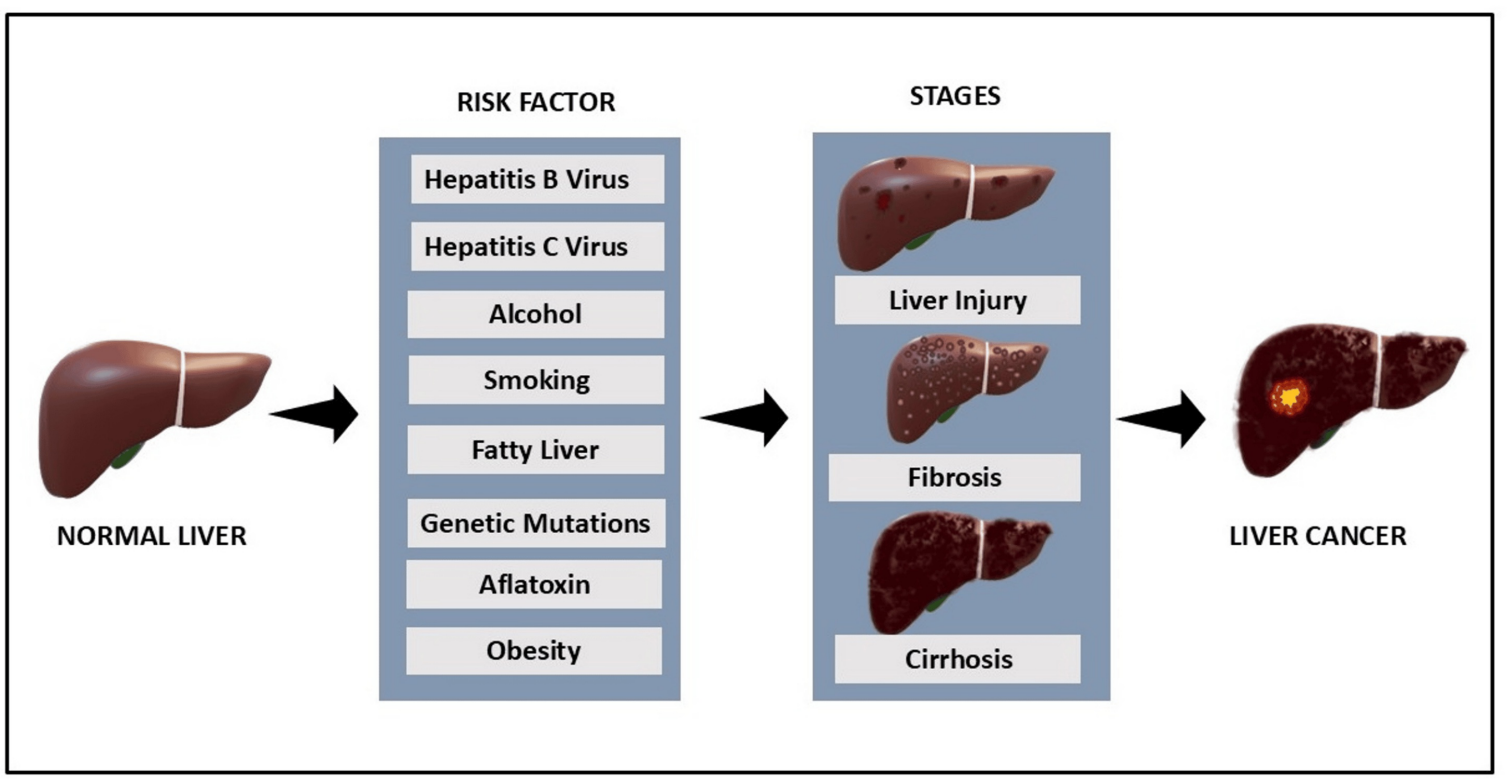
Risk factors in liver disease progression from healthy liver to hepatocellular carcinoma This image has been created by the authors using Microsoft PowerPoint software (Microsoft Corp., Redmond, WA).

This figure shows the progression of liver disease from a normal liver to HCC. Key risk factors like viral infections, alcohol, obesity, etc. drive this process, leading to liver injury, fibrosis, and cirrhosis [31]. The final stage is HCC, where chronic damage results in liver cancer. The diagram highlights the crucial role of these risk factors in liver disease development (Figure 1).

Fatty Liver Disease

Metabolic dysfunction-associated fatty liver disease was first proposed by an international panel of experts in 2020 to better reflect the role of metabolic factors in liver disease progression; MAFLD focuses on metabolic dysfunction (e.g., obesity, insulin resistance, and type 2 diabetes) as the primary drivers of liver disease. This definition acknowledges that MAFLD focuses on metabolic dysfunction (e.g., obesity, insulin resistance, and type 2 diabetes) with or without the influence of alcohol. Earlier, fatty liver disease was classified into NAFLD and alcoholic liver disease (ALD), based on alcohol consumption. The prevalence of MAFLD is expected to be similar to NAFLD due to the overlapping metabolic conditions. This updated classification aims to improve early detection, management, and treatment of liver diseases by emphasizing metabolic health, addressing the complexities of fatty liver diseases, and ultimately reducing the risk of HCC. Early identification of high-risk patients through non-invasive biomarkers, lifestyle interventions, and pharmacological treatments is crucial to reduce the growing burden of liver cancer associated with fatty liver diseases [32, 33].

Fatty liver diseases, both NAFLD and AFLD, are significant risk factors for HCC, the most common form of liver cancer [34]. Globally, NAFLD affects 24%-25% of the population, with higher rates in regions like South America and the Middle East, driven by the rising prevalence of obesity, type 2 diabetes, and metabolic syndrome [35, 36]. Non-alcoholic fatty liver disease can progress from simple steatosis (fat accumulation without inflammation) to NASH, which is characterized by liver inflammation and damage. NASH significantly increases the risk of liver fibrosis, cirrhosis, and HCC. Approximately 20% of NASH patients progress to cirrhosis, and about 1% develop HCC, even in the absence of cirrhosis [37]. The incidence of liver cancer is rising globally, correlating with the growing rates of obesity and NAFLD. A liver biopsy is the gold standard for diagnosing NASH. However, non-invasive techniques such as magnetic resonance imaging (MRI), ultrasound elastography, serum biomarkers, etc. have also been developed [38, 39].

Alcohol Consumption

In patients with alcohol-associated cirrhosis, the annual incidence of HCC varied from 0.9% to 5.6% [8]. In 2019, alcohol was linked to about one-fifth of HCC-related fatalities worldwide [40]. Designated as a Group 1 carcinogen by the IARC, alcohol is linked to HCC and various other cancers, including cancers of the mouth, throat, esophagus, breast, colon, rectum, and larynx [41]. Prolonged and excessive alcohol intake can result in conditions such as fatty liver, acute/chronic hepatitis, and cirrhosis, substantially increasing the relative risk of developing HCC by three to 10 times [41].

Hepatitis B and C Viral Infections

A significant percentage of HCC cases are caused by the oncoviruses HBV and HBC, while the function of the HDV in the development of liver carcinogenesis is yet unknown [42, 43]. Viral proteins that destabilize cellular signaling pathways and induce prolonged liver inflammation with a compromised antiviral immune response are common methods employed by HBV, HCV, and HDV to cause hepatocarcinogenesis [44]. Hepatitis B infection is characterized by deoxyribonucleic acid (DNA) integration, promoting genomic instability, while an HCV infection is responsible for metabolic reprogramming, resulting in steatosis [42]. Many factors, including HBV gene integration, mutation-induced genomic instability, and activation of cancer-promoting signaling pathways, have been implicated in the pathogenesis of HBV-associated HCC [45]. Hepatitis B virus-HCC involves complex interactions across multiple genes and steps where various cancer-promoting mechanisms accelerate disease evolution from inflammation to tumorigenesis [46]. Hepatitis B vaccination is expected to prevent HCC by preventing both an initial infection and the establishment of a chronic carrier condition [47]. Research on the progression of HBV-HCC continues to uncover new mechanisms such as epigenetics, exosomes, autophagy, metabolic regulation, and immune suppression. The severity in patients with HBV infection can vary, and those with underlying cirrhosis are at an increased risk of developing advanced stages of HCC [48]. Direct-acting antivirals (DAAs) have demonstrated a durable virologic response in 95% of cases recently [49]. With its tremendously high cure rates and stellar safety profile, DAA therapy has completely changed the way HCV is treated [50]. Although HCV infection is linked to hepatocellular carcinoma, the exact mechanism of the relationship between the virus and the tumor is yet unknown [51]. Research on the HCV replication system is essential to our comprehension of both acute and chronic HCV infections. Hepatitis B virus and HCV infections are major risk factors for HCC worldwide, and with increasing control of HBV through vaccination and improved access to treatment, viral hepatitis-related PLC rates may decrease in the future [52, 53]. Unfortunately, a decrease in PLC rates may be offset by increasing levels of obesity and its metabolic complications and increasing alcohol consumption [54].

Other Predisposing Factors

As a genotoxic hepatocarcinogen, aflatoxin B1 (AFB1) is believed to cause cancer by forming DNA adducts that change the genetic composition of liver cells [55]. Its metabolism by microsomal cytochrome enzymes leads to the formation of AFB1-exo-8,9-epoxide, which is considered responsible for its genotoxic effects [56]. However, there is increasing evidence that oxidative stress caused by AFB1 may also play a significant role in its genotoxic effects [56,57]. Obesity affects over 650 million individuals worldwide and is a well-established risk factor for HCC [58]. The mechanisms linking obesity and HCC correlate with adipose tissue remodeling, alteration in the gut microbiome, genetic factors, endoplasmic reticulum stress, oxidative stress, and epigenetic changes [59, 60]. Approximately 40% of patients with liver disease have a history of smoking, while an increasing number of studies are exploring the potential impact of smoking on chronic liver diseases [61]. A recent research study has found an association between smoking and the development of HCC, identifying carcinogenic compounds like 4-aminobiphenyl as significant risk factors for liver cancer [62]. Patients with diabetes mellitus have a two- to three-times higher occurrence rate of HCC [63]. Genetic mutations play a key role in HCC, often triggered by chronic viral infections (hepatitis B or C), alcohol use, and aflatoxin exposure. Common mutations include the TP53 gene, which regulates the cell cycle, and alterations in cell signaling pathways like Wnt/β-catenin and RAS-RAF-MAPK. These changes drive uncontrolled cell growth and tumor development. Understanding these mutations is critical for creating targeted therapies and improving patient outcomes [64].

Diagnostic techniques

Hepatocellular carcinoma is an exceptionally severe and fatal form of cancer, marked by high morbidity and mortality [65]. Most of the time, patients with HCC have no early symptoms, so it is crucial to diagnose it in its early stages [66]. Diagnostic techniques play a pivotal role in detecting the disease at an early stage. 

The histology of liver cancer is a critical component of diagnostic techniques, particularly in distinguishing early-stage HCC from more advanced cases. According to the WHO and international consensus guidelines, HCC is classified into early and progressed stages based on tumor size and the degree of cellular differentiation [66]. Early HCC, typically less than 2 cm with poorly defined margins, is commonly associated with cirrhotic livers and is characterized by hypovascularity. In contrast, progressed HCC presents with diverse macroscopic forms, such as nodular, massive, or diffuse patterns [67]. Key histological features of HCC include well-vascularized tumors, broad trabecular or pseudoglandular structures, cytologic atypia, and a loss of the reticulin network [66]. Recognizing these distinctions is essential for accurate diagnosis, staging, and management, enabling more effective and tailored treatment strategies for liver cancer.

Various invasive and non-invasive diagnostic techniques are used for the diagnosis of liver cancer.

Invasive Techniques

Biopsy is an invasive technique that has numerous advantages over imaging techniques for diagnosing HCC [67]. A small tissue sample is collected through biopsy, which provides vital information, particularly for diagnosing well-differentiated HCC [68]. Biopsy samples can be obtained through various methods, such as the liquid and tissue biopsy methods.

A liquid biopsy is a less invasive method of analyzing a sample of blood or other body fluids to detect cancer cells or fragments of tumor DNA. This technique analyzes circulating tumor cells, cell-free DNA, non-coding RNAs, and extracellular vesicles in the blood [69]. Liquid biopsy is a supplementary tool, offering the potential for HCC detection, identification of minimal residual disease, treatment selection, and monitoring of therapeutic responses [70]. This field of liquid biopsy is a rapidly evolving field that is transforming cancer care by enabling non-invasive, real-time tumor monitoring [71].

Tissue biopsy is a procedure in which a small piece of tissue is removed from the body using a needle, endoscope, or surgical method and examined under a microscope. The result helps doctors make informed decisions about treatment options. Tissue biopsy remains an invaluable tool in cancer diagnosis [72]. Analyzing tumor samples through this method provides crucial information for determining the presence and nature of cancer [73]. A tissue biopsy can expand our understanding of cancer biology, identify novel biomarkers, and guide the development of new therapies [71]. However, tissue biopsy is not without risks, including morbidity from complications such as pain and bleeding, particularly in patients with cirrhosis. The incidence of minor complications is around 5.9%, and significant hemorrhage occurs in 0.5% of cases [74].

Ultimately, both liquid and tissue biopsies have important roles to play in the diagnosis and management of HCC [74]. Tissue biopsies may become pivotal in matching patients to appropriate treatments, while liquid biopsies are likely to play complementary roles in early HCC detection and monitoring of treatment response [75].

Non-invasive Techniques

Non-invasive diagnostic techniques are the cornerstone in the early detection and monitoring of HCC, minimizing patient discomfort while providing essential diagnostic information. Biopsies of HCC are rarely required nowadays, as non-invasive techniques have become the preferred approach. Ultrasound, computed tomography (CT), and MRI are the most commonly used diagnostic techniques [76].

When it comes to HCC screening and monitoring, diagnosis, staging, and evaluating therapy response, imaging is essential [77]. Imaging techniques, such as USG, CT scans, MRI, and positron emission tomography (PET), play a crucial role in the surveillance, diagnosis, staging, and post-treatment monitoring of HCC [78]. These advanced imaging modalities provide valuable information to guide clinical decision-making and patient management [79]. A standardized classification system called Liver Imaging Reporting and Data System (LI-RADS) is used to interpret liver imaging, particularly in high-risk patients, and complements histopathological and clinical data for accurate diagnosis and staging. It assists in the distinction of benign from malignant lesions and helps direct treatment decisions [80].

Ultrasonography is a widely used real-time imaging technique that utilizes high-frequency sound waves to create images of internal organs, tissues, and blood flow, helping doctors diagnose and monitor liver diseases, including HCC [81]. The American Association for the Study of Liver Diseases (AASLD) and the European Association for the Study of the Liver (EASL) both recommend ultrasound as a standard monitoring technique for patients at risk of developing HCC. This includes patients with cirrhosis, chronic HBV infection with risk factors (older age, male gender, family history), chronic HCV infection with advanced fibrosis or cirrhosis, and individuals with NAFLD who have advanced fibrosis [82]. The availability of innovative ultrasound contrast agents, such as Levovist and Sonazoid (particularly in Japan), has significantly improved the diagnostic and treatment approaches for HCC [83]. These contrast-enhanced ultrasound techniques allow for better characterization of liver lesions, improved detection of small tumors, and more accurate guidance for treatment planning [84]. The advancements in sonographic technologies have transformed the clinical management of HCC, enabling enhanced treatment guidance and potentially improving the prognosis for HCC patients.

Computed tomography scan refers to a computerized X-ray imaging procedure that is extensively used for accurate diagnosis and treatment planning, which forms the backbone of any medical therapy [85]. It provides detailed images of the entire liver through multiphase sequential scans. Traditionally, contrast-enhanced single-slice helical CT has been considered a reliable tool in diagnosing and staging HCC [78]. However, "new, advanced CT imaging" techniques, such as angiographic maps of the key vascular structures, have the potential to provide even more improved diagnostic capabilities [86]. These emerging technical developments in CT imaging, including perfusion CT, dual-energy CT, and hepatocyte-specific contrast imaging, have constituted one of the greatest breakthroughs in diagnostic radiology [87]. These technologies enhanced the ability to diagnose accurately and stage HCC, ultimately informing and guiding the most appropriate treatment approach.

Magnetic resonance imaging offers several key advantages when comparing MRI to its main competitor, CT scan [88]. Most notably, MRI does not expose the patient to ionizing radiation, which is a significant benefit compared to the radiation exposure from CT scans [89]. Additionally, it provides superior tissue contrast, allowing for enhanced visualization and characterization of liver lesions [90]. It also offers the flexibility of not always requiring the use of contrast agents, and when contrast is needed, smaller volumes are typically used compared to CT scans [91]. Furthermore, MRI can utilize hepatocyte-specific contrast agents, which can provide additional diagnostic information [92]. A comprehensive liver MRI examination typically includes a range of advanced imaging techniques. The T2-weighted imaging technique detects regions of fluid, inflammation, or edema, while the T1-weighted imaging technique highlights various tissue types to provide precise anatomical information. Diffusion-weighted imaging and the apparent diffusion coefficient mapping technique help to detect tumors by measuring water molecule movement. Contrast-enhanced imaging helps to detect and evaluate liver cancers, liver function, and anomalies in blood flow more accurately by improving the image of blood vessels and blood supply [93]. These techniques allow for a thorough assessment of liver anatomy, tissue characteristics, and vascular features [94]. Additionally, more recent advances in MRI technology have expanded the scope of liver imaging, now enabling the quantification of liver fat, iron content, and even the assessment of liver stiffness through MR elastography [95]. Dynamic contrast-enhanced imaging techniques can also provide valuable information about liver perfusion and thermodynamics [96]. Overall, the versatility and diagnostic capabilities of MRI make it a powerful tool for the evaluation and management of HCC.

Positron emission tomography is now considered a significant invention; PET uses the glucose analog 2-18F-fluoro-2-deoxy-D-glucose (FDG), as cancer cells have a higher rate of glucose consumption. The metabolism of FDG is similar to that of glucose, but upon phosphorylation by hexokinase, it becomes metabolically trapped and accumulates within the cancer cell. The PET imaging system differentiates between normal and malignant tissues by quantifying the distribution of FDG within the body [97]. While PET scanning is highly sensitive for picking up extrahepatic metastatic disease, its lower sensitivity for the primary hepatic lesion may not provide a complete picture for initial HCC staging [98]. Therefore, PET-CT should be considered as a complementary tool, combined with other recommended modalities like CT, MRI, and bone scintigraphy, to formulate the optimal treatment plan for HCC patients, especially those being evaluated for potentially curative options like hepatic resection or liver transplantation. The metabolic information from PET can provide valuable insights that enhance the staging and management of HCC [99].

Laboratory diagnostic technique

The role of serum α-fetoprotein (AFP) in diagnosing HCC is controversial; certain clinical guidelines still recommend it as a biomarker [100]. Studies have reported that AFP can influence the ability of cancer cells to maintain and propagate their undifferentiated, stem-like state [101]. Serum AFP is a protein produced by the fetal liver and yolk sac during pregnancy. It declines after birth and remains at low levels throughout life. Serum AFP was the first known tumor marker, identified in the blood of patients with liver cancer and certain other cancers. Elevated AFP levels are associated with a poorer prognosis in liver cancer patients. High AFP levels can indicate more advanced disease. Serum AFP has been found useful in selecting candidates for liver transplantation and predicting the risk of tumor recurrence after liver surgery [102]. This suggests AFP may play a crucial role in promoting the differentiated, stem-like characteristics of HCC cells. Furthermore, numerous studies have demonstrated the tumor-promoting effects of AFP, which include inhibition of apoptosis, stimulation of proliferation, and enhancement of migration, invasion, and metastasis [103]. Importantly, AFP has also been shown to serve as a crucial prognostic biomarker for patients with HCC undergoing immunotherapy, highlighting its multifaceted roles in HCC biology and clinical management [104].

These diagnostic techniques are commonly used for HCC screening due to their non-invasive nature and widespread availability; they may have limitations in detecting small or early-stage lesions [105]. Similarly, CT scans can provide detailed images of liver lesions but may struggle to differentiate HCC from other liver conditions [106]. Serum biomarkers, such as AFP, have been traditionally used for HCC surveillance and diagnosis, but they lack specificity and sensitivity, particularly in the early stages of the disease [107, 108]. Some advancements in imaging technology, biomarker research, and non-invasive diagnostic techniques have led to the development of more accurate and sensitive methods for early HCC detection and diagnosis. By integrating multi-dimensional data, clinicians can enhance patient outcomes and tailor personalized treatment strategies for individuals with HCC. This approach can facilitate early detection and timely, customized interventions, ultimately improving survival rates and quality of life for patients with HCC.

Unfortunately, HCC is often detected at advanced stages, hindered by the limitations of early diagnostic techniques, resulting in poor patient outcomes. When it is diagnosed in an advanced stage, it often results in limited treatment options and a poor prognosis [109]. It also diminishes the potential for curative treatments like surgical resection or liver transplantation [110]. Advanced-stage HCC is more likely to have spread to other parts of the body, reducing the effectiveness of therapies [111]. Consequently, patients face reduced survival rates and diminished quality of life [112].

Ongoing research on emerging imaging techniques, next-generation sequencing, multi-omics analysis, terahertz spectroscopy, metabolomics, epigenetic biomarkers, and microbiome analysis holds promise for future early diagnosis of HCC [112]. These methods will provide non-invasive ways to assess tumor genetics, metabolism, and molecular alterations, offering valuable insights into tumor heterogeneity, treatment response, and disease progression [113]. Ultimately, these insights can improve diagnosis and enable more treatment strategies. Liquid biopsy techniques, including circulating tumor DNA analysis, circulating tumor cell detection, and extracellular vesicle analysis, will continue to advance in their ability to assess tumor genetic alterations and monitor disease progression in real-time [114]. Next-generation sequencing will enable even more comprehensive genomic profiling of HCC tumors, identifying a wider range of genetic mutations, copy number variations, and gene fusions that drive cancer development. Many of the mutated genes still need to be understood in terms of their molecular functions and roles in cancer, offering a significant opportunity for future research [115, 116]. Terahertz spectroscopy, as a non-ionizing imaging technique, will further demonstrate its potential in the early diagnosis of digestive cancers like HCC by distinguishing between normal and cancerous tissues based on their unique absorption properties [117]. Metabolomic profiling of body fluids will identify more disease-specific metabolic signatures in HCC, with aberrant metabolites serving as potential diagnostic biomarkers [118]. Analysis of epigenetic alterations, such as DNA methylation patterns and non-coding RNAs, will provide even deeper insights into the development of HCC [119]. Characterizing the gut microbiome and its impact on liver health will offer new perspectives on HCC risk and enable the development of more advanced microbiome-based diagnostic tools [61].

Collectively, these emerging technologies and multi-omic approaches will significantly enhance the early detection, accurate diagnosis, and personalized treatment of HCC, ultimately improving patient outcomes.

## Conclusions

Liver cancer remains a significant global health challenge, characterized by its high incidence and mortality rates. The global burden of liver cancer is expected to remain substantial, highlighting the need for continued focus on prevention, early detection, and improved treatment strategies. This review highlights the complex nature of liver cancer, encompassing its various types, epidemiological trends, risk factors, and the critical importance of early and accurate diagnosis. Thus, continuous work and invention are necessary to enhance the current diagnostic resources deployed. In conclusion, advancing early diagnosis and personalized treatment strategies is essential for effectively reducing the global burden of liver cancer.
